# Ischemic Acute Kidney Injury Perturbs Homeostasis of Serine Enantiomers in the Body Fluid in Mice: Early Detection of Renal Dysfunction Using the Ratio of Serine Enantiomers

**DOI:** 10.1371/journal.pone.0086504

**Published:** 2014-01-29

**Authors:** Jumpei Sasabe, Masataka Suzuki, Yurika Miyoshi, Yosuke Tojo, Chieko Okamura, Sonomi Ito, Ryuichi Konno, Masashi Mita, Kenji Hamase, Sadakazu Aiso

**Affiliations:** 1 Department of Anatomy, Keio University School of Medicine, Shinanomachi, Shinjuku-ku, Tokyo, Japan; 2 Graduate School of Pharmaceutical Sciences, Kyushu University, Maidashi, Higashi-ku, Fukuoka, Japan; 3 Innovative Science Research and Development Center, Shiseido Co., Ltd., Fukuura, Kanazawa-ku, Yokohama, Japan; 4 Department of Pharmacological Sciences, International University of Health and Welfare, Kitakanemaru, Ohtawara, Tochigi, Japan; University of Florida, United States of America

## Abstract

The imbalance of blood and urine amino acids in renal failure has been studied mostly without chiral separation. Although a few reports have shown the presence of D-serine, an enantiomer of L-serine, in the serum of patients with severe renal failure, it has remained uncertain how serine enantiomers are deranged in the development of renal failure. In the present study, we have monitored serine enantiomers using a two-dimensional HPLC system in the serum and urine of mice after renal ischemia-reperfusion injury (IRI), known as a mouse model of acute kidney injury. In the serum, the level of D-serine gradually increased after renal IRI in parallel with that of creatinine, whereas the L-serine level decreased sharply in the early phase after IRI. The increase of D-serine was suppressed in part by genetic inactivation of a D-serine-degrading enzyme, D-amino acid oxidase (DAO), but not by disruption of its synthetic enzyme, serine racemase, in mice. Renal DAO activity was detected exclusively in proximal tubules, and IRI reduced the number of DAO-positive tubules. On the other hand, in the urine, D-serine was excreted at a rate nearly triple that of L-serine in mice with sham operations, indicating that little D-serine was reabsorbed while most L-serine was reabsorbed in physiological conditions. IRI significantly reduced the ratio of urinary D−/L-serine from 2.82±0.18 to 1.10±0.26 in the early phase and kept the ratio lower than 0.5 thereafter. The urinary D−/L-serine ratio can detect renal ischemia earlier than kidney injury molecule-1 (KIM-1) or neutrophil gelatinase-associated lipocalin (NGAL) in the urine, and more sensitively than creatinine, cystatin C, or the ratio of D−/L-serine in the serum. Our findings provide a novel understanding of the imbalance of amino acids in renal failure and offer a potential new biomarker for an early detection of acute kidney injury.

## Introduction

D-Serine is *de novo* synthesized from its enantiomer, L-serine, in mammals [1] and has a pivotal role in glutamatergic neurotransmission in the central nervous system (CNS) [2]. In the peripheral organs, the physiological role or regulation of D-serine remains largely unknown, apart from its regional control in the kidney. D-Serine in the plasma stems from dietary uptake and also from tissues that express the synthetic enzyme of D-serine, and it is excreted by the kidneys for the most part into the urine. Although an uptake carrier of serine in pars recta of renal proximal tubules has a low stereospecificity, only a small portion of filtered D-serine is reabsorbed since L-serine, overwhelming D-serine in primitive urine, competitively inhibits the uptake of D-serine [3]. The reabsorbed D-serine is metabolized by tubular D-amino acid oxidase (DAO) into hydroxypyruvate, hydrogen peroxide, and ammonia. Therefore, the kidney is thought to keep the plasma D-serine at a low level, up to 3% in total plasma serine in humans [4]–[6].

Several links between deranged D-serine regulation and renal dysfunction have been reported: plasma D-serine level increases up to more than 20% of total serine in patients with highly elevated plasma creatinine [4]–[6], a high level of DAO is detected in the urine of patients with chronic renal failure [7], and renal ischemia-reperfusion injury (IRI) reduces activity of renal DAO in rats [8]. D-Serine is also known to selectively damage the pars recta of proximal tubules in rats, leading to aminoaciduria and glucosuria [9], [10]. Therefore, D-serine has been regarded as both an indicator and an exacerbating factor of renal dysfunction. However, how D-serine is deranged in renal dysfunction remains uncertain.

To monitor alterations of serine enantiomers in the development of renal dysfunction, we used a two-dimensional HPLC (2D-HPLC) system. In the present study, using mice with renal ischemia-reperfusion injury (IRI) as a model of acute kidney injury (AKI), we report that disposition of D-serine in the body fluid after renal IRI is closely correlated with that of creatinine. The alteration of serum D-serine originates from loss of renal DAO activity and reduced glomerular filtration rate (GFR). We also demonstrate that ratios of serine enantiomers in the casual urine may serve as a sensitive biomarker in the early detection of AKI.

## Materials and Methods

### Ethics Statement

All experiments on animals were carried out in accordance with institutional guidelines. The study protocol was approved by the Animal Experiment Committee of KEIO University.

### Materials

The enantiomer of serine and HPLC-grade acetonitrile were obtained from Nacalai Tesque (Kyoto, Japan). Methanol of HPLC grade, trifluoroacetic acid (TFA), citric acid monohydrate, and boric acid were purchased from Wako (Osaka, Japan). Water was purified using a Milli-Q gradient A 10 system (Millipore, Bedford, MA, USA). All other reagents for 2D-HPLC were of the highest reagent grade and were used without further purification.

### Human Samples

Human serum samples were obtained from BioServe Biotechnologies (Beltsville, MD, USA), and consisted of four male healthy donors and four male patients with severe renal failure. All of them were Caucasian. None of them had smoking habits or were afflicted with diabetes mellitus. Further clinical information, e.g., the stage of renal failure or pathological diagnosis, was not available. The donors’ ages and renal function parameters are listed in Table S1.

### Animals

Animals were maintained in a specific pathogen-free environment, housed in a light-controlled room with a 12-h light/dark cycle, and allowed ad libitum access to food and water. C57BL/6J mice were purchased from CLEA Japan (Tokyo, Japan). A mouse line with a C57BL/6J background lacking DAO activity systemically with a natural point mutation of Gly-181-Arg was generated by backcrossing ddY/DAO^-^ mice with C57BL/6J, as described previously [11]. Global serine racemase (SR)-knockout mice were generated as reported previously [12].

### IRI Model

Male mice between 12–16 weeks of age underwent experimental procedures for the IRI model. Before induction of IRI, the right kidneys were removed through a small flank incision under pentobarbital. After 12 days, these mice were randomized into two groups: sham-operated (sham-op) control and IRI. The mice were anesthetized with pentobarbital, and the left kidneys were exposed through a small flank incision. Blood flow through the left renal artery and vein was interrupted with a nontraumatic clamp (Schwartz Micro Serrefines; Fine Science Tools Inc., Vancouver, Canada). After 45 min of ischemia, the vessel clamp was removed. The return of the original surface color of the kidneys was confirmed visually, and the abdomen was closed in layers. In sham-operated control mice, the kidneys were treated identically, except for clamping. At 4, 8, 20, or 40 h after reperfusion, mice were anesthetized with diethyl ether; blood and urine were collected from the inferior vena cava and bladder, respectively; and then kidneys were removed with or without perfusion fixation, depending on the subsequent experimental procedure. Sera were separated in a BD microtainer (BD, Franklin Lakes, NJ, USA) by centrifugation at 1500×g for 10 min. The levels of creatinine (Cr) and blood urea nitrogen (BUN) in the sera or urine were determined using a Fuji DRI-CHEM 4000 system (FujiFilm, Tokyo, Japan). Serum cystatin C as well as urinary kidney injury molecule-1 (KIM-1) and neutrophil gelatinase-associated lipocalin (NGAL) were quantified using mouse ELISA kits from R&D Systems (Minneapolis, MN, USA).

### Two-dimensional HPLC

Serine enantiomers were determined using a 2D-HPLC system, as previously reported [11], [13]. Briefly, amino acids in the serum were derivatized with 4-fluoro-7-nitro-2,1,3-benzoxadiazole (NBD-F) (Tokyo Kasei, Tokyo, Japan); subjected to HPLC (NANOSPACE SI-2 series, Shiseido, Tokyo, Japan); separated into each amino acid by a reversed-phase column (a microbore-monolithic ODS column, 0.53 mm ID×1000 mm, provided by Shiseido); and further separated into enantiomers by an enantioselective column (Sumichiral OA-2500S, 1.5 mm ID×250 mm, self-packed; material was obtained from Sumika Chemical Analysis Service, Osaka, Japan). The fluorescence intensity was detected at 530 nm with excitation at 470 nm. Representative chromatograms of 2D separation of authentic D−/L-serine and those in mouse serum and urine are shown in Supplementary Fig. S1.

### Histological Analysis

Mice were anesthetized with diethyl ether and perfused transcardially with ice-cold phosphate buffer (PB, pH 7.4) and subsequently with 2% paraformaldehyde in PB. Tissues were then cryoprotected in a 20% sucrose solution in PB at 4°C until they sank. They were frozen in Tissue-Tek O.C.T. Compound (Sakura Finetek Japan, Tokyo, Japan). Sections 10 µm thick were sliced on a cryostat at −19°C and stored at −80°C until they were used.

Sections were rinsed in phosphate buffer-saline (PBS, pH 7.4), stained with hematoxylin and eosin (H & E), dehydrated, cleared, and mounted with Entellan new (Merck, Darmstadt, Germany).

For fluorescence staining, sections were rinsed in phosphate buffer-saline (PBS, pH 7.4) and incubated in 20 µg/ml fluorescein-labeled *Lotus tetragonolobus* lectin (LTL) in PBS for 30 min at room temperature. The sections were washed in PBS and transfered into a DAO-activity staining solution [7 mM pyrophosphate buffer (pH 8.3), 0.1% horseradish peroxidase (Sigma-Aldrich, St. Louis, MO, USA), Cy3-conjugated tyramide (1∶400; Perkin-Elmer, Waltham, MA, USA), 0.065% sodium azide, 0.6% nickel ammonium sulfate, 22 mM D-proline, 20 µM FAD] [11], and incubated at room temperature for 7 min under dark conditions. They were washed in PBS and mounted using ProLong Gold Antifade Reagent with DAPI (Invitrogen, Carlsbad, CA, USA).

Sections labeled with fluorescence were imaged using a Zeiss LSM 510 confocal microscope (Carl Zeiss, Oberkochen, Germany). Each section being compared was imaged under identical conditions.

### Enzyme Activity Assay of DAO

The activity of DAO was determined as described previously [11]. Briefly, 50 µl of tissue lysate was added to a mixture [150 µl of 100 mM D-alanine, 100 µl of 0.1 mM flavin adenine dinucleotide (FAD), 150 µl of 700 units/ml catalase in 133 mM sodium pyrophosphate (pH8.3), and 50 µl of 70% v/v MeOH], processed with constant agitation at 37°C for 30–60 min, and terminated by adding 500 µl of 10% trichloroacetic acid. To 250 µl of the supernatant solution were added 250 µl of 5 M KOH and 250 µl of 0.5% 4-amino-3-hydrazino-5-mercapto-1,2,4-triazole in 0.5 M HCl. After 15 min incubation at room temperature, 250 µl of 0.75% KIO_4_ in 0.2 M KOH was added to the mixture with vigorous shaking, and absorbance at 550 nm was measured. DAO activity was calculated as described by Watanabe et al. [14] and expressed as the amount of D-alanine oxidized per minute per milligram of protein.

### Statistical Analysis

All values in the text and figures of this study indicate means ± standard error of mean (SEM). Statistical analyses for the experiments were performed with a two-tailed Student’s *t*-test or one-way ANOVA followed by Tukey’s multiple comparison test, in which *P*<0.05 was assessed as significant. All analyses were performed using Prism 5 (GraphPad Software, La Jolla, CA, USA).

## Results

The D-serine in body fluid is maintained physiologically at low micromolar levels, and its quantitative measurement requires a highly sensitive and selective technique. We measured serine enantiomers using a 2D-HPLC system that enabled us to detect D−/L-serine ranging from 1 fmol to 100 pmol quantitatively with chiral selectivity. Our system was sensitive enough to detect the elevation of D-serine in the serum of patients with severe renal failure (RF) (Fig. S2), and our results were comparable to previous reports [4]–[6].

### Derangement of Serum Serine Enantiomers after Renal IRI

**Figure 1:**
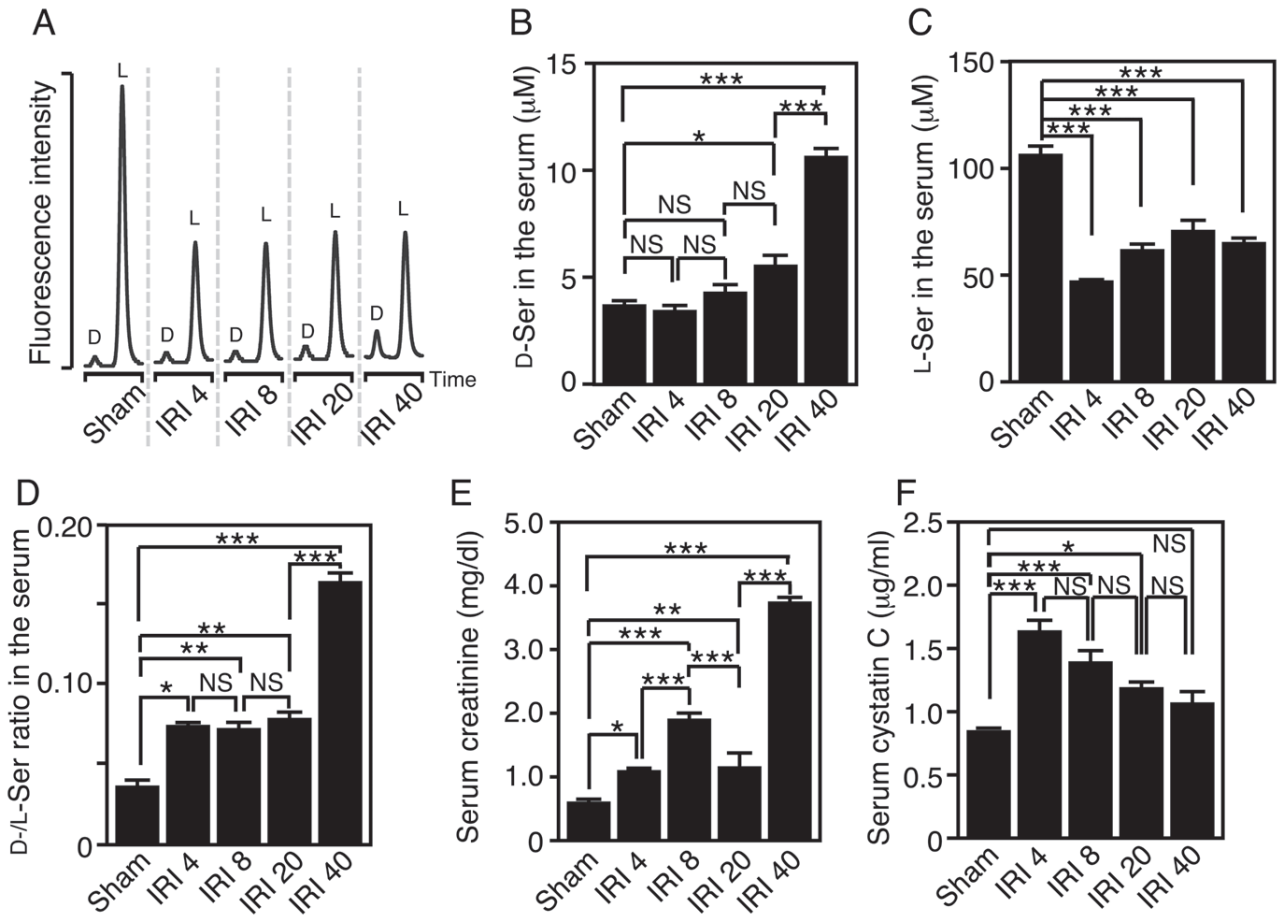
Renal IRI increases D-serine and reduces L-serine in mouse serum. (A) Shown are typical chromatograms of serum D−/L-serine obtained by 2D-HPLC. [Mice with sham-op (Sham); and those at 4, 8, 20, and 40 h after renal IRI (IRI 4, IRI 8, IRI 20, and IRI 40)] (B-F) Concentrations of serum D-serine (B), L-serine (C), creatinine (E), and cystatin C (F) in the C57BL/6J mice were determined, and ratios of D-serine to L-serine concentrations were calculated (D) (Sham, n = 8; IRI 4, n = 5; IRI 8, n = 9; IRI 20, n = 6; and IRI 40, n = 7). *P<0.05, **P<0.01, ***P<0.001 (one-way ANOVA followed by Tukey’s multiple comparison test). NS means ‘not significant’. Data are plotted as the mean ± SEM.

To understand whether such alterations of serine enantiomers might reflect renal dysfunction, we generated renal IRI mice as an experimental model of AKI. Using the 2D-HPLC system, we quantified levels of serine enantiomers in these mice. In C57BL/6J mice, the serum D-serine did not change significantly at 4 or 8 h, was elevated at 20 h, and showed a further increase at 40 h after reperfusion (Sham, 3.7±0.3 µM; IRI 4, 3.4±0.3 µM; IRI 8, 4.3±0.4 µM; IRI 20, 5.5±0.5 µM; IRI 40, 10.6±0.4 µM) (Fig. 1A and B). On the other hand, the level of serum L-serine plunged at 4 h and remained low thereafter (Sham, 106.1±5.0 µM; IRI 4, 46.9±0.9 µM; IRI 8, 61.5±5.6 µM; IRI 20, 70.6±7.5 µM; IRI 40, 64.7±2.2 µM) (Fig. 1A and C). The ratio of D−/L-serine rose at 4 h due to the reduction of L-serine and further increased at 40 h after reperfusion (Sham, 0.036±0.004; IRI 4, 0.074±0.005; IRI 8, 0.073±0.009; IRI 20, 0.082±0.009; IRI 40, 0.164±0.008) (Fig. 1D). Similarly, the levels of serum creatinine fluctuated between 1.0–2.0 mg/dl from 4 h to 20 h and were elevated at 40 h after reperfusion (Sham, 0.59±0.05 mg/dl; IRI 4, 1.108±0.04 mg/dl; IRI 8, 1.89±0.09 mg/dl; IRI 20, 1.14±0.22 mg/dl; IRI 40, 3.73±0.09 mg/dl) (Fig. 1E). Unlike D-serine and creatinine, serum cystatin C, a marker for early detection of renal dysfunction, surged at 4 h and thereafter decreased gradually (Sham, 0.84±0.01 µg/ml; IRI 4, 1.63±0.08 µg/ml; IRI 8, 1.39±0.09 µg/ml; IRI 20, 1.19±0.05 µg/ml; IRI 40, 1.06±0.10 µg/ml) (Fig. 1F). These results show that the level of serum D-serine parallels that of serum creatinine and that a reduction in serum L-serine detects early renal dysfunction, as does an increase of serum cystatin C.

### Mechanism Underlying Derangement of Serum Serine Enantiomers through SR and DAO

**Figure 2:**
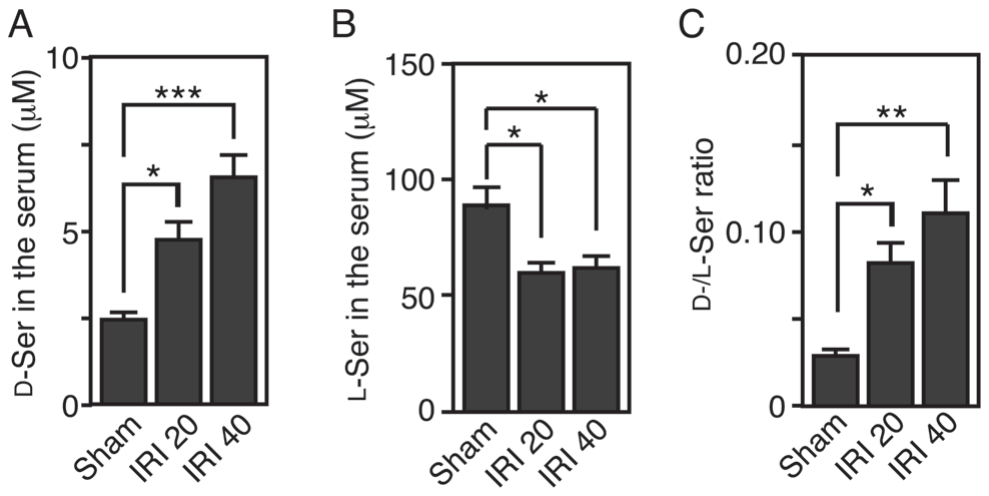
Knockout of SR does not affect alterations of serine enantiomers after IRI. Concentrations of D-serine (A), L-serine (B), and D−/L-serine ratio (C) in the sera of SR-KO mice were determined using 2D-HPLC (Sham, n = 4; IRI 20, n = 4; and IRI 40, n = 4). *P<0.05, **P<0.01, ***P<0.001 (one-way ANOVA followed by Tukey’s multiple comparison test). Data are plotted as the mean ± SEM.

Regulation of serum serine enantiomers is not well understood, but it is known to be kept in balance by production, degradation, uptake, and excretion. Because SR converts L-serine into D-serine [1] and its expression in the kidney had been reported [15], we next tested whether SR contributes to the derangement of serine enantiomers after IRI. Serine enantiomers in SR knockout (SR-KO) mice were determined using 2D-HPLC. Disruption of SR reduced the basal level of serum D-serine (C57BL/6J wild-type, 3.7±0.3 µM; SR-KO, 2.5±0.2 µM). However, in the same manner as the alterations detected in C57BL/6J wild-type mice, the levels of serum D-serine increased progressively at 20 and 40 h after IRI (Sham, 2.5±0.2 µM; IRI 20, 4.8±0.5 µM; IRI 40, 6.6±0.6 µM) (Fig. 2A), while serum L-serine was significantly reduced at 20 h and remained low at 40 h after reperfusion (Sham, 89.6±9.6 µM; IRI 20, 60.0±4.5 µM; IRI 40, 62.1±5.8 µM) (Fig. 2B). The D−/L-serine ratio was elevated parallel to the increase of D-serine (Sham, 0.029±0.004; IRI 20, 0.082±0.012; IRI 40, 0.110±0.018) (Fig. 2C). These results indicate that SR does not trigger the derangement of serine enantiomers caused by renal IRI.

**Figure 3:**
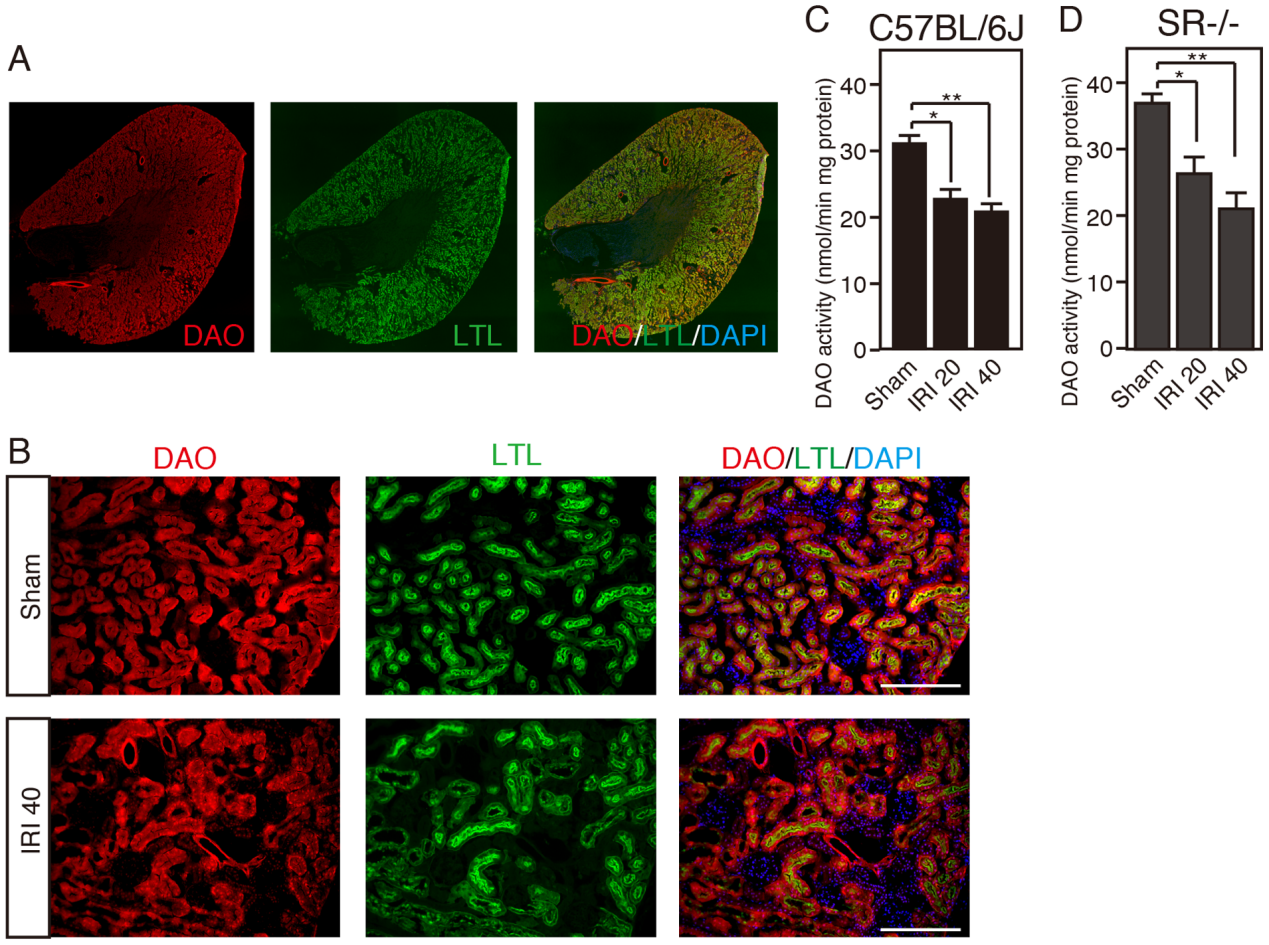
IRI reduces the number of proximal epithelial cells with DAO activity. (A and B) A horizontally sliced section of the kidney in a C57BL/6J wild-type mouse (A) and high magnifications of renal cortex in the mice (Sham or IRI 40) (B) were stained with DAO enzyme histochemistry, a proximal tubular marker (LTL), and a nuclear marker (DAPI). Scale bars, 200 µm. (C and D) DAO activity in the total kidneys of C57BL/6J wild-type mice [Sham, n = 4; IRI 20, n = 5; and IRI 40, n = 6] (C) and SR-KO mice [Sham, n = 3; IRI 20, n = 4; and IRI 40, n = 3] (D) was determined in a quantitative assay. *P<0.05, **P<0.01 (one-way ANOVA followed by Tukey’s multiple comparison test). Data are plotted as the mean ± SEM.

**Figure 4:**
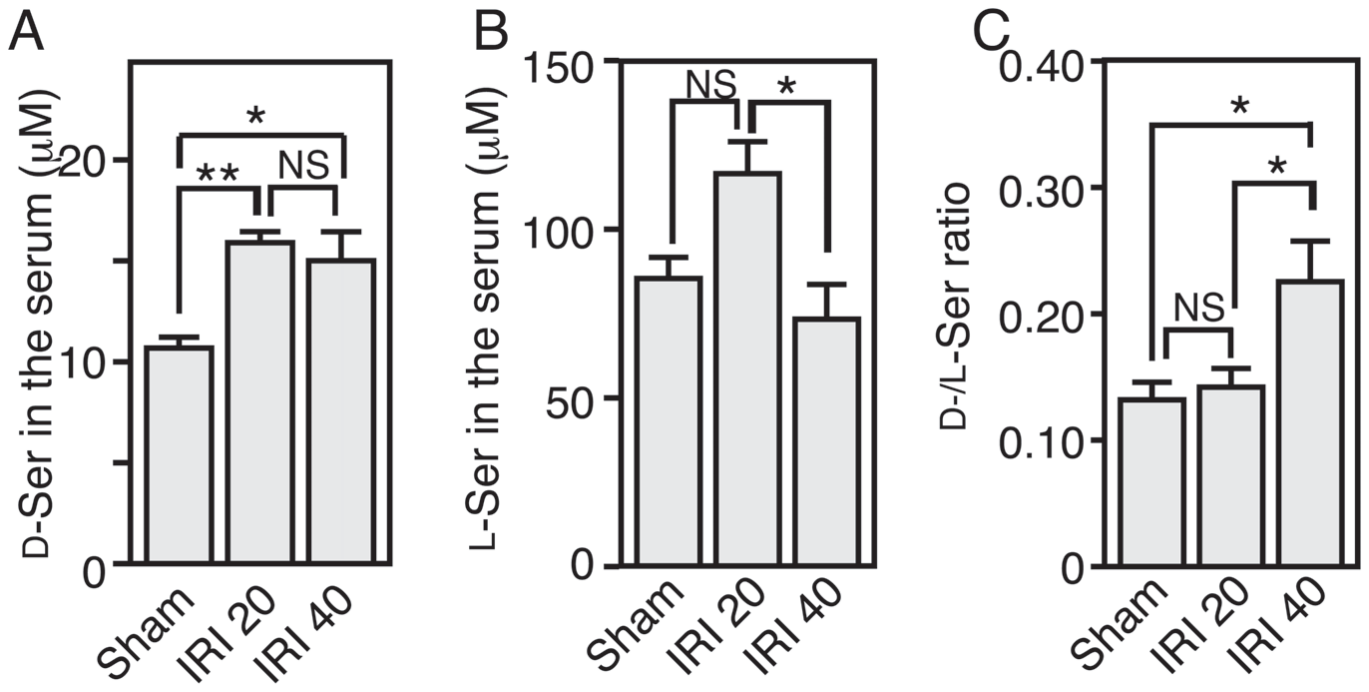
Lack of DAO activity suppresses IRI-induced accumulation of D-serine in serum. (A-C) The serine enantiomers in the sera of DAO-null mice were determined using 2D-HPLC, and their concentrations (D-serine, A; L-serine, B) and ratio (C) are shown (Sham, n = 6; IRI 20, n = 6; and IRI 40, n = 5). *P<0.05, **P<0.01 (one-way ANOVA followed by Tukey’s multiple comparison test). NS is ‘not significant’. Data are plotted as the mean ± SEM.

Serum level of D-serine can be influenced by DAO, a unique degrading enzyme of D-serine, highly expressed in the kidney. Enzyme activity of endogenous renal DAO was detected in frozen tissue sections using enzyme histochemistry (EHC), as described previously [11], [16]. DAO enzymatically labeled with Cy3 was colocalized with FITC-labeled LTL, a proximal tubule marker (Fig. 3A and B). DAO was detected in LTL-positive proximal tubular epithelial cells in sham-op controls, and IRI reduced the number of proximal tubules with DAO activity at 40 h after reperfusion (Fig. 3B). In a quantification assay, DAO enzyme activity in the total kidney of C57BL/6J wild-type mice was suppressed to 73% and 67% of that of sham-op controls at 20 and 40 h after IRI, respectively [*F*_(2, 12)_ = 10.97, *P* = 0.0020] (Fig. 3C). Reduced renal DAO enzyme activity was detected also in SR-KO mice after IRI (71% at 20 h and 57% at 40 h compared with sham-op controls)[*F*_(2, 7)_ = 11.34, *P* = 0.0064] (Fig. 3D). To confirm the effect of reduced DAO activity on serum levels of serine enantiomers, we measured serum D−/L-serine in mice lacking DAO activity with a point mutation of G181R (DAO-null mice). Lack of DAO increased the basal level of serum D-serine (C57BL/6J wild-type, 3.7±0.3 µM; DAO-null, 10.8±0.4 µM). In the DAO-null mice, IRI elevated the serum D-serine at 20 h, but did not show a further increase at 40 h after reperfusion (Sham, 10.8±0.4 µM; IRI 20, 16.0±0.6 µM; IRI 40, 15.1±1.5 µM) [*F*_(2, 15)_ = 8.866, *P* = 0.0029] (Fig. 4A). Levels of serum L-serine tended to increase at 20 h and were reduced at 40 h after reperfusion (Sham, 85.5±5.8 µM; IRI 20, 116.6±10.0 µM; IRI 40, 73.3±11.1 µM) [*F*_(2, 15)_ = 5.832, *P* = 0.0134] (Fig. 4B). The ratio of D−/L-serine was unchanged at 20 h and elevated at 40 h after reperfusion compared with sham-op (Sham, 0.132±0.012; IRI 20, 0.142±0.013; IRI 40, 0.225±0.032) [*F*_(2, 15)_ = 5.776, *P* = 0.0138] (Fig. 4C). These results show that lack of DAO activity inhibits the steady increase of serum D-serine or decrease of serum L-serine caused by IRI, suggesting that the loss of DAO activity contributes, at least in part, to the derangement of serine enantiomers.

### Impact of Loss of DAO activity on Renal Pathology after IRI

**Figure 5:**
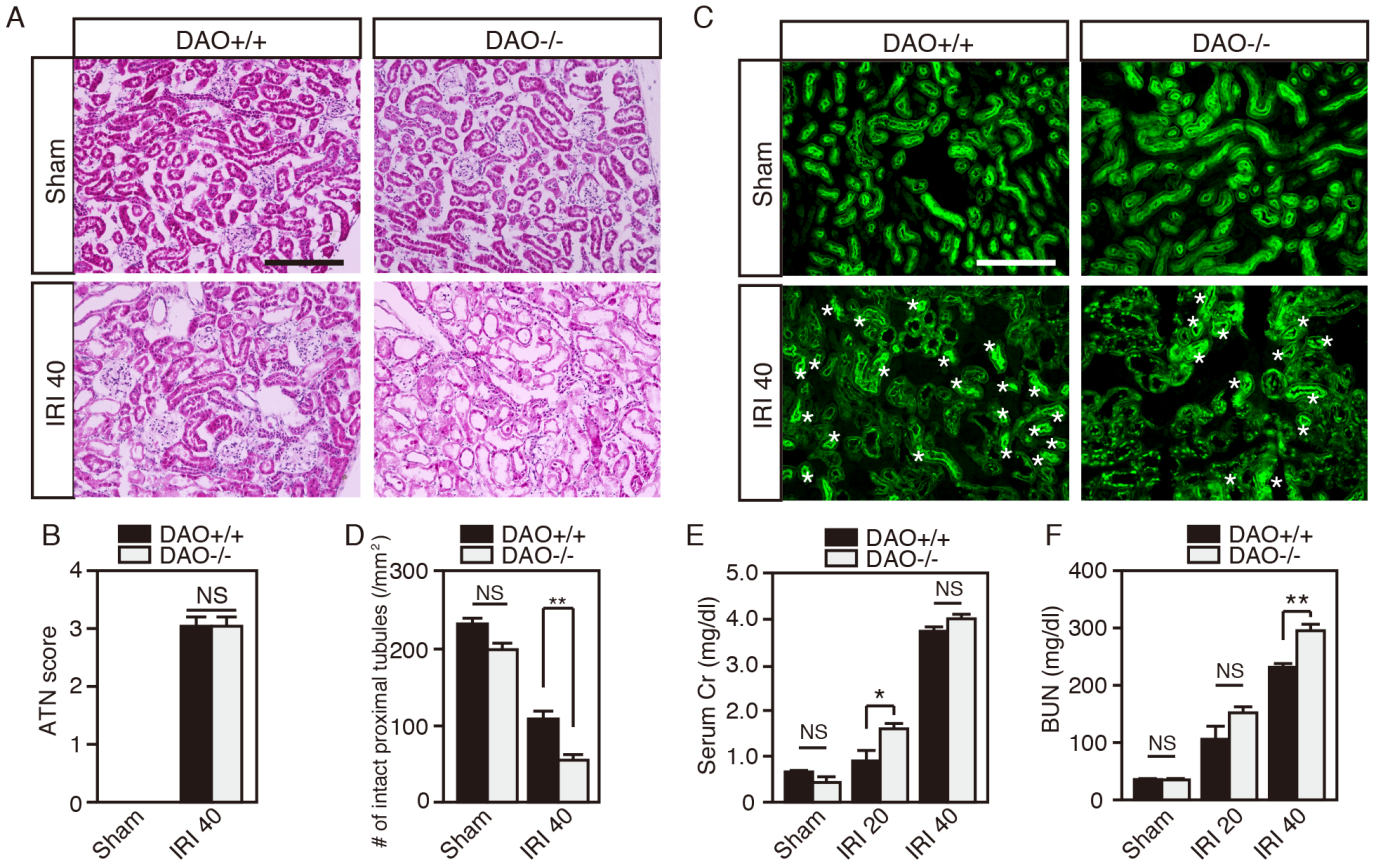
Lack of DAO activity exacerbates loss of intact proximal tubules and renal dysfunction induced by IRI. (A and C) Renal cortices in C57BL/6J wild-type (DAO^+/+^) and DAO-null mice (DAO^−/−^) after sham-op (Sham) or IRI (at 40 h after reperfusion) were stained with H & E (A) or LTL (C). (B) Damaged tubules were evaluated and ATN score was calculated in slices stained with H & E (n = 5, each group). (D) Number of intact proximal tubules was counted in slices stained with LTL (n = 5, each group). (E and F) Serum Cr (E) and BUN (F) in DAO^+/+^ [Sham n = 5; IRI 20, n = 5; and IRI 40, n = 7] and DAO^−/−^ mice [Sham, n = 6; IRI 20, n = 5; and IRI 40, n = 6] were measured. *P<0.05, **P<0.01, and NS is ‘not significant’ (one-way ANOVA followed by Tukey’s multiple comparison test). Data are plotted as the mean ± SEM.

In rats, D-serine is nephrotoxic because of its metabolites produced by DAO. To further evaluate the contribution of DAO to the progression of renal damage, we performed histological analysis of the kidney in C57BL/6J wild-type mice and DAO-null mice with or without IRI. The sham-op DAO-null mice showed no obvious difference in the pattern of tubules compared with sham-op wild-type controls as shown in H & E staining (Fig. 5A, upper panels). IRI tended to damage tubules more severely in DAO-null mice than in wild-type animals (Fig. 5A, lower panels) (percentage of damaged tubules: wild-type, 51.7±5.3%; and DAO-null, 65.7±5.8%. n = 5, each), although the ATN score did not differ significantly between wild-type and DAO-null mice (Fig. 5B). The number of intact proximal tubules, which was evaluated using staining with LTL, was significantly lower in DAO-null mice than in wild-type animals after renal IRI (Fig. 5C and D) [Fig. 5D: *F*_(3, 16)_ = 89.64, *P*<0.0001]. The increase of serum Cr and BUN triggered by IRI tended to be higher in DAO-null mice than in wild-type controls, with significance at reperfusion time of 20 h for Cr and 40 h for BUN (Fig. 5E and F) [Fig. 5E: *F*_(5, 28)_ = 159.4, *P*<0.0001; Fig. 5F: *F*_(5, 28)_ = 89.3, *P*<0.0001]. These results indicated that DAO protects proximal tubules mildly. Therefore, the D-serine metabolites produced by DAO do not seem to exacerbate IRI-induced renal dysfunction in mice, but undegraded D-serine is suggested to do so.

### Urinal Excretion of Serine Enantiomers after Renal IRI

**Figure 6:**
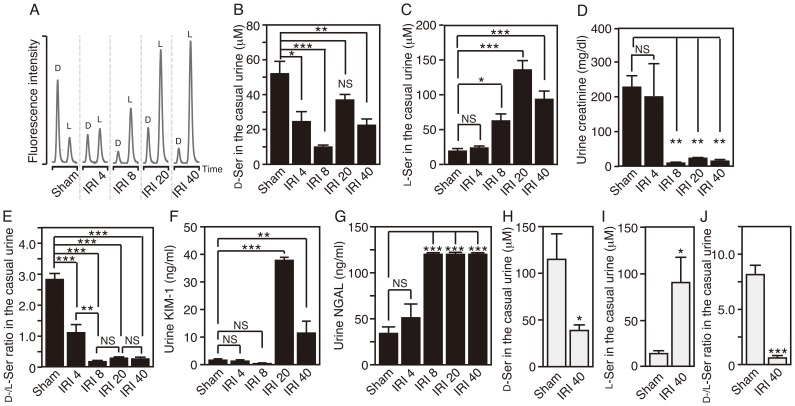
IRI inverts D−/L-serine ratio in urine. Urinary serine enantiomers were analyzed using 2D-HPLC. (A) Typical chromatograms showing urinary D−/L-serine in C57BL/6J wild-type mice with or without renal IRI. (B-G) Concentrations of D-serine (B), L-serine (C), creatinine (D), KIM-1 (F), and NGAL (G), and ratios of D−/L-serine (E) in the urine of the wild-type mice were determined (Sham, n = 7; IRI 4, n = 5; IRI 8, n = 5; IRI 20, n = 5; and IRI 40, n = 5). *P<0.05, **P<0.01, ***P<0.001 (one-way ANOVA followed by Tukey’s multiple comparison test). NS means ‘not significant’. (H-J) Concentrations of D-serine (H) and L-serine (I), and D−/L-serine ratios (J) in the urine of DAO-null mice were determined. *P<0.05, ***P<0.001 (two-tailed Student’s *t* test). Data are plotted as the mean ± SEM.

Since loss of DAO activity in mice did not nullify the effect of IRI on serine enantiomers, involvement of other factors, such as failure to excrete serine enantiomers in the urine, cannot be excluded. Amounts of D−/L-serine in the urine display a pattern that is the inverse of those in the serum (Fig. 1A and 6A, *see chromatograms of sham-op groups*). Urinary excretion of D-serine is nearly three times greater than that of L-serine, due to preferential reabsorption of L-serine in the proximal tubule in physiological conditions. IRI reduced by half the concentration of D-serine in the casual urine at 4 h after reperfusion, with the concentration fluctuating at low levels thereafter (Sham, 52.0±7.6 µM; IRI 4, 24.5±5.7 µM; IRI 8, 9.9±1.1 µM; IRI 20, 36.9±3.3 µM; IRI 40, 22.4±3.8 µM) [*F*_(4, 22)_ = 9.288, *P* = 0.0001] (Fig. 6A and B). On the other hand, excretion of L-serine in the casual urine began to increase from 8 h and remained high at 20 and 40 h after reperfusion (Sham, 19.0±3.0 µM; IRI 4, 23.6±2.7 µM; IRI 8, 62.6±9.9 µM; IRI 20, 136.1±14.9 µM; IRI 40, 93.8±12.1 µM) [*F*_(4, 22)_ = 29.49, *P*<0.0001] (Fig. 6A and C). Concentrations of solutes in casual urine are often expressed in terms of urinary creatinine because the excretion of creatinine is constant across individuals. However, creatinine correction is not reliable in renal dysfunction because creatinine excretion itself is disturbed in severe renal dysfunction (Fig. 6D). In this case, the D−/L-serine ratio is reliable for evaluating the urinary excretion of serine enantiomers because the value is not affected by the level of urine concentration. The ratio of D−/L-serine showed a sharp decline at 4 h and became lower than 0.5 after 8 h (Sham, 2.82±0.18; IRI 4, 1.10±0.26; IRI 8, 0.16±0.01; IRI 20, 0.28±0.02; IRI 40, 0.25±0.04) [*F*_(4, 22)_ = 62.33, *P*<0.0001] (Fig. 6E). On the other hand, the levels of urinary KIM-1, a key molecule for early diagnosis of AKI [17], surged at 20 h, but did increase significantly at 4 and 8 h after IRI (Sham, 1.7±0.6 ng/ml; IRI 4, 1.3±0.5 ng/ml; IRI 8, 0.4±0.2 ng/ml; IRI 20, 37.8±1.1 ng/ml; IRI 40, 11.4±4.3 ng/ml) [*F*_(4, 23)_ = 81.45, *P*<0.0001] (Fig. 6F). Urinary levels of NGAL, another promising molecule [18], displayed an upward tendency at 4 h, but with no statistical significance, and rose significantly at 8 h after IRI and thereafter (Sham, 34.3±7.3 ng/ml; IRI 4, 51.1±16.3 ng/ml; IRI 8, 119.6±1.2 ng/ml; IRI 20, 119.2±1.5 ng/ml; IRI 40, 119.3±1.5 ng/ml) [*F*_(4, 23)_ = 31.89, *P*<0.0001] (Fig. 6G). These results show that the urinary D−/L-serine ratio detects renal ischemia earlier than serum creatinine, urinary KIM-1, or NGAL, and more sensitively than serum cystatin C. And such deranged excretion of serine enantiomers caused by renal IRI may be the main trigger for both accumulation of D-serine and reduction of L-serine in the serum.

DAO is detected in the urine of chronic renal failure, so we further tested whether DAO affects the drop in the urinary D−/L-serine ratio after renal IRI. In DAO-null mice, IRI strikingly reduced the urinary D−/L-serine ratio (Fig. 6H–J). Therefore, non-metabolic factors such as disturbed reabsorption in the proximal tubule may contribute to the decline in the urinary D−/L-serine ratio after IRI.

## Discussion

We have shown that renal IRI perturbs homeostasis of serine enantiomers in the serum and urine. In the serum, D-serine accumulates gradually in the late phase after IRI, whereas the level of L-serine falls sharply in the early phase. In the casual urine, the concentration of D-serine is reduced in the early phase after IRI, while that of L-serine is elevated in the late phase. Reduced activity of renal DAO caused by IRI is in part responsible for D-serine accumulation in the serum. Decreased GFR and disturbed proximal reabsorption are suggested to contribute to a large extent to such alterations of serine enantiomers.

The serum level of D-serine is physiologically retained at up to 5 µM, less than 1/20 of that of L-serine, and has little individual variability in humans (Fig. S2) [19], [20] or in mice (Fig. 1) [21]. The degree of D-serine accumulation, corresponding to 10–20% of L-serine, in the blood of mice with severe renal dysfunction after renal IRI is similar to that in humans with RF (Fig. 1 and S2) [4]–[6], validating this animal model for investigating serine derangement in renal dysfunction. The level of serum D-serine is balanced by food intake, metabolism by SR/DAO, and renal filtration rate. For two reasons, renal reabsorption has little effect on the serum D-serine level under physiological conditions. One is preferential uptake of L-serine by a serine transporter on proximal tubules under the condition that L-serine dominates D-serine in primitive urine [3]. The other is degradation of reabsorbed D-serine by DAO in the proximal tubules. When activity of renal DAO is reduced due to the damage on the proximal tubules, disturbed degradation of reabsorbed D-serine increases its influx into systemic circulation. On the other hand, in DAO-null mice, reabsorbed D-serine passes through proximal tubules without degradation by DAO, returns back to systemic circulation, and elevates its levels in the serum. Because DAO activity is absent regardless of renal damage, IRI-induced increase of D-serine is attenuated in DAO-null mice (Fig. 4A). Our finding that loss of DAO or SR in mice could not completely nullify the IRI-induced alteration of D-serine in the serum (Fig. 2A and 4A) indicates that decreased GFR, but not impaired reabsorption, may play a role in D-serine accumulation caused by IRI. This view is supported by our results that the serum D-serine level correlates well with that of serum creatinine (Fig. 1B and E), which is not reabsorbed in tubules. The decrease of D-serine concentration in casual urine after renal IRI parallels that of urinary creatinine (Fig. 6B and D), indicating that the decrease can be explained by reduced renal filtration rate and concentrating ability, but not by impaired proximal reabsorption or degradation by DAO (Fig. 6H-J). Thus, disposition of D-serine in body fluid resembles that of creatinine.

On the other hand, disposition of L-serine is different from that of D-serine or creatinine. Although in this study we did not investigate the renal metabolism of L-serine, it is natural to think that the alterations of L-serine result largely from impairment of proximal reabsorption because the decrease in the serum (Fig. 1C) and increase in the urine (Fig. 6C) occur in the acute phase after renal IRI. In DAO-null mice, such serum L-serine reduction is attenuated although concentration of urinary L-serine is increased as in wild-type mice (Fig. 4B and 6I). Decrease of GFR caused by more-severe renal damage in DAO-null mice than in wild-type mice (Fig. 5E) may reduce urine volume and total urinary L-serine excretion, which might explain the attenuation of L-serine loss in the serum.

D-Serine potentially involves renal pathophysiology. Although high-dose injection of D-serine damages proximal tubules through oxidative stress due to metabolism by DAO in rats [9], [10], [22], [23], our results suggest that D-serine metabolism by DAO does not exacerbate IRI-induced renal pathology in mice, but rather improves it (Fig. 5). D-Serine can affect *N*-methyl-D-aspartate (NMDA) glutamate receptors in the kidney. The NMDA receptor is a ligand-gated ion channel that belongs to a large family of ionotropic glutamate receptors and requires binding of a coagonist besides glutamate for its full activation. D-Serine is now regarded as an endogenous coagonist of NMDA receptors in the CNS [2], [24]. In addition to broad distribution of NMDA receptors in the CNS, it has become evident that functional NMDA receptors are also expressed in various parts of nephrons including the glomeruli, proximal tubules, and collecting ducts, indicating their diverse involvement in the regulation of renal function [25]–[30]. Moreover, several inhibitors of NMDA receptors have been reported to ameliorate the progression of renal dysfunction in rats with renal IRI [31], [32]. Considering that D-serine potentiates NMDA-evoked currents in renal cell culture [25], derangement of serum D-serine after renal IRI may affect the progression of renal pathology through impairing activities of NMDA receptors in the nephrons.

Renal IRI is a common cause of AKI, occurring with hypotension and cardiovascular surgery and inevitably during kidney transplantation. AKI is a critical clinical condition associated with a high degree of mortality, even with best supportive care. In clinical practice, AKI is predominantly detected by changes in serum creatinine. However, because serum creatinine increases only after substantial loss of GFR, the current clinical diagnosis of AKI based on creatinine limits its early detection, and consequently the prompt implementation of preventive measures, in routine clinical care. Recently, numerous new biomarkers – categorized as inflammatory mediators, excreted tubular proteins, and surrogate markers that indicate tubular damage – have been proposed as early detection markers of AKI [33]. Because AKI is multifactorial and heterogeneous in origin, it seems likely that not one single marker but a panel of biomarkers will be required to detect all subtypes of AKI. We have newly shown that the D−/L-serine ratio, especially in casual urine, detects early-stage ischemic AKI more sensitively than other promising biomarkers. Since the urinary D−/L-serine ratio is mostly determined by the rate of proximal reabsorption of L-serine, reduction of the ratio represents damage specific to proximal tubules. The reproducibility of our results in human AKI and their clinical significance, such as diagnostic specificity/sensitivity and prognostic value of the D−/L-serine ratio, should be verified in future studies.

We conclude from our study that ischemic AKI disrupts the physiological balance of serine enantiomers in the serum and urine. The imbalance of amino acids as total amounts of D- and L-forms has been well characterized in chronic RF [34], [35], and the supplementation of lost amino acids is considered important in supportive therapy. Considering that D- and L-serine are imbalanced differently in AKI, the imbalance of total amino acids in chronic RF should also be reevaluated by separating D- and L-amino acids. Thus, our study provides a novel understanding of serine enantiomers in ischemic AKI as well as a new paradigm of amino acid balance in renal physiology and pathology.

## Supporting Information

Figure S1 **Determination of serine enantiomers by 2D-HPLC.** (A-J) Chromatograms show representative 2D-separation of authentic D−/L-serine as their NBD derivatives (A and B), or those in the serum (C-F) or urine (G-J) in wild-type mice with sham-operation (C, D, G, and H) or IRI (E, F, I, and J). (A, C, E, G, and I) Reversed-phase separation of NBD-serine was performed by using a microbore ODS column. The black bars indicate the fractions online collected to a loop and transferred to the enantioselective column, Sumichiral OA-2500S, in which NBD-serine enantiomers were further separated (B, D, F, H, and J).
(TIF)

Figure S2 **Serum serine enantiomers are altered in patients with renal failure.** D- (A) and L-serine (B) in the serum of healthy volunteers (n = 4) and patients with RF (n = 4) were measured with 2D-HPLC. (C) The ratio of D-serine to L-serine is shown. *P<0.05, **P<0.01, ***P<0.001 (Student’s *t* test).
(TIF)

Table S1 **Ages and parameters for renal function in human samples.** Data are shown as mean ± S.E.M. (N = 4, each).
(PDF)
