# Supplementary figures and images for: Ischemic Acute Kidney Injury Perturbs Homeostasis of Serine Enantiomers in the Body Fluid in Mice: Early Detection of Renal Dysfunction Using the Ratio of Serine Enantiomers
Source: PLoS One. 2014 Jan 29;9(1):e86504. doi: 10.1371/journal.pone.0086504 (PMC3906037; doi:10.1371/journal.pone.0086504)

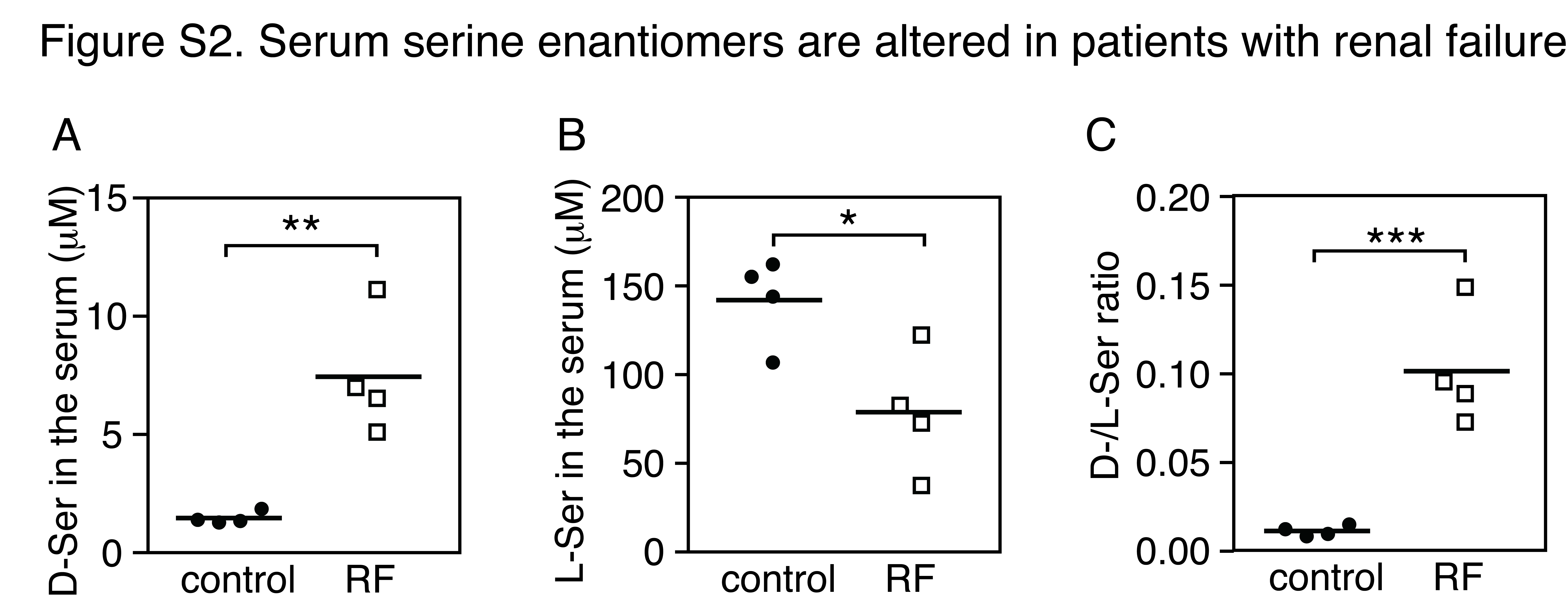

Supplement: Figure S2 — Serum serine enantiomers are altered in patients with renal failure. D- (A) and L-serine (B) in the serum of healthy volunteers (n = 4) and patients with RF (n = 4) were measured with 2D-HPLC. (C) The ratio of D-serine to L-serine is shown. *P<0.05, **P<0.01, ***P<0.001 (Student’s t test). (TIF) [file pone.0086504.s002.tif]
