# Supplementary material for: Ischemic Acute Kidney Injury Perturbs Homeostasis of Serine Enantiomers in the Body Fluid in Mice: Early Detection of Renal Dysfunction Using the Ratio of Serine Enantiomers
Source: PLoS One. 2014 Jan 29;9(1):e86504. doi: 10.1371/journal.pone.0086504 (PMC3906037; doi:10.1371/journal.pone.0086504)
Supplement: Table S1 — Ages and parameters for renal function in human samples. Data are shown as mean ± S.E.M. (N = 4, each). (PDF) [file pone.0086504.s003.pdf]

Table S1. Ages and parameters for renal function in human samples

	Healthy control	Renal failure
Age	50.0 ± 0	53.0 ± 7.4
Serum Cr (mg/dl)	1.0 ± 0.1	11.1 ± 1.2
BUN (mg/dl)	15.6 ± 0.4	60.0 ± 8.8

Data are shown as mean ± S.E.M. (N = 4, each).
